# The effects of perceived teacher support on online mathematics learning power: the mediating roles of artificial intelligence literacy and cognitive tools

**DOI:** 10.3389/fpsyg.2026.1763924

**Published:** 2026-02-24

**Authors:** Yaping Hu, Liying Liu, Hua Yang, Xuelong Jia

**Affiliations:** College of Science, Tianjin University of Science and Technology, Tianjin, China

**Keywords:** AI literacy, cognitive tools, higher education, mediating role, online mathematics learning power, perceived teacher support

## Abstract

With the rapid advancement of generative artificial intelligence (GenAI), teaching and learning in higher education are undergoing profound transformations. As a foundational competency for students in the digital age, pathways to foster online mathematics learning power require exploration. Although teacher support is recognized as a critical factor, the mechanisms through which it might foster students’ new digital competencies, thereby contributing to online mathematics learning power within the context of intelligent technologies remain underexplored. This study aims to construct a multiple mediation model to examine how perceived teacher support predicts online mathematics learning power through two pathways: AI literacy (the ability to leverage artificial intelligence for mathematical cognition) and the use of cognitive tools (e.g., MATLAB, GeoGebra). A questionnaire survey was conducted among 758 undergraduates enrolled in online mathematics courses at a comprehensive university in eastern China. The instruments included scales for perceived teacher support, AI literacy, cognitive tools, and online mathematics learning power. Data were analyzed using structural equation modeling and bootstrap sampling to examine direct and mediating effects. The results confirm that within the generative artificial intelligence context, perceived teacher support directly predicts students’ online mathematics learning power while also indirectly predicts it by fostering AI literacy and cognitive tools proficiency. This reveals the mechanisms linking environmental support, digital competencies, and learning outcomes. This study suggests that educators adopt a teaching strategy integrating direct support, AI literacy cultivation, and cognitive tool guidance. This entails incorporating AI literacy and cognitive tools training into online mathematics course design within supportive learning environments. Doing so can effectively develop student competencies and prepare them for the intelligent era.

## 1 Introduction

The rapid advancement of generative artificial intelligence (GenAI) is driving profound transformations in higher education teaching models and learning methods, establishing online learning as a mainstream educational format (Bennett et al., 2020; Xiao et al., 2020; Abe, 2020). As the core capacity for learners to acquire knowledge, develop abilities, and achieve growth in digital environments, online learning power has garnered increasing scholarly attention (Broadbent and Poon, 2015; Kember, 2009; Wang and Wu, 2022; Wang, 2022). This power, shaped by the interplay of internal factors (e.g., digital literacy, adaptability, learning attitudes; Prior et al., 2016) and external factors (e.g., teacher support, peer interaction, learning context; Kedia and Mishra, 2023), has emerged as a critical focus of scholarly inquiry.

With its highly logical and abstract nature, mathematics requires greater clarity of reasoning, timely feedback, and concrete conceptual understanding (Wadsworth et al., 2007). When these discipline-specific requirements intersect with the online learning environment, students often face significant challenges, drawing scholarly attention to the composition and influencing factors of online mathematics learning power (Aviory et al., 2025; Bringula et al., 2021). However, empirical research on effectively improving this competency remains scarce in China, making it crucial to investigate the underlying mechanisms. Extensive research has established digital literacy and AI literacy as critical foundations for academic achievement (Theobald et al., 2021; Almogren et al., 2024; Mohamed et al., 2025), and cognitive tools are also vital for mathematics learning (e.g., Feng, 2024). Hence, how teachers foster these competencies to influence online mathematics learning power remains a key question to be explored.

This study develops and tests a multiple mediation model to clarify the pathways and mechanisms through which perceived teacher support predicts students’ online mathematics learning power. It makes three key contributions. First, it integrates social cognitive theory with digital competence literature to propose a multi-path framework linking environmental support, AI literacy, cognitive tools, and learning power, thereby revealing how external support translates into internal capability development in AI-era online mathematics learning. Second, it operationalizes the general concept of digital literacy into the domain-specific construct of “AI literacy” in mathematics learning contexts, while specifying that “technological tools” denotes subject-specific cognitive tools such as MATLAB and GeoGebra. This enhances the disciplinary relevance and instructional applicability of the research. Finally, methodologically, it employs serial mediation analysis to compare the predictive strengths of AI literacy and cognitive tools within the mediation pathways and to investigate how their combined effect contributes to online mathematics learning power, providing empirical evidence to guide teaching practice.

## 2 Literature review

### 2.1 Online mathematics learning power

Online mathematics learning power represents an extension and discipline-specific evolution of traditional learning power theory (Crick, 2006; Crick et al., 2013) in the digital age. Grounded in the double helix theory of learning (McGettrick, 2002) and classical frameworks of online learning power (Broadbent and Poon, 2015; Kember, 2009), it is defined as the systemic capability essential for learners to succeed in mathematics learning within online environments. This encompasses key dimensions such as the active construction of mathematical knowledge, strategic problem-solving for complex issues, the ability to engage in rigorous logical reasoning, and the sustained development of deep mathematical thinking. The formation of this capability is influenced by both individual and environmental factors. Individual factors include learning motivation, self-regulatory skills, and digital literacy (Crick et al., 2015), while environmental factors encompass learning platform design, teacher support, and peer interaction (Kember, 2009). Although the academic community has not reached a consensus on the dimensional structure of online learning power, core elements such as adaptation, cognition, application, and reflection have gained broad recognition (Crick, 2007; Zimmerman, 2002; Artino Jr, 2012; Zheng et al., 2025). The synergistic development of these core abilities helps learners achieve continuous growth in complex and dynamic online environments (Broadbent, 2017). Based on the characteristics of the mathematics discipline, this study conceptualizes online mathematics learning power through four key dimensions: adaptability in mathematics learning, cognitive capability in mathematics learning, applicative ability in mathematics learning, and reflective capacity in mathematics learning, thereby establishing the theoretical analytical framework.

### 2.2 AI literacy

Generative artificial intelligence (GenAI) tools, capable of assuming multiple roles such as teacher, tutor, clerk, and designer, are reshaping the forms and boundaries of student learning and transforming how students learn (Dwivedi et al., 2023). By providing personalized content delivery, instant feedback, and immersive learning environments, these tools stimulate and support learners’ capacity for autonomous exploration and continuous development (Liu et al., 2025). In response to this transformation, learners require corresponding artificial intelligence literacy (Burgsteiner et al., 2016), defined as the ability to understand fundamental AI knowledge and concepts. Long and Magerko (2020), from a human-computer interaction perspective, proposed an AI literacy framework comprising 17 elements, encompassing aspects such as knowing AI, understanding AI, and using AI. Building upon this, Heyder and Posegga (2021) structured AI literacy into three conceptual blocks: functional AI literacy, critical AI literacy, and socio-cultural AI literacy. Ng et al. (2021), drawing on Bloom’s Taxonomy of Educational Objectives, developed a coding framework for core AI competencies through a literature review, identifying four key areas for cultivating AI literacy: knowing and understanding AI, using and applying AI, evaluating and creating AI, and AI ethics. Wang et al. (2023), based on a “technology-cognition-ethics” model, constructed a framework for core AI literacy across four dimensions: awareness, use, evaluation, and ethics. Chiu et al. (2024) proposed a framework consisting of five key components: technology, impact, ethics, collaboration, and self-reflection. United Nations Educational, Scientific and Cultural Organization (UNESCO) has defined the core of AI literacy as knowledge, understanding, skills, and values. Its further elaborated specific competency framework covers multiple dimensions, including a human-centered approach, AI ethics, technology and application, and system design, aiming to provide theoretical support for global AI literacy education.

In the context of higher education, student AI literacy has emerged as a prominent research focus (Dai et al., 2020; Laupichler et al., 2022; Wang et al., 2023). While a unified definitional framework for artificial intelligence literacy is yet to be established, existing research in this area spans numerous dimensions, including awareness, knowledge, skills, thinking modes, attitudes, ethics, values, evaluation, creation, and socio-cultural elements. Generative AI not only assists students in acquiring knowledge diversely and efficiently to enhance learning capacity (Chiu et al., 2023) but also directly supports the development of core competencies such as critical thinking (Long and Magerko, 2020), self-directed learning (Dai et al., 2020), and innovative creation (Ng et al., 2021). Consequently, for online mathematics learning, AI literacy fundamentally reshapes students’ problem-solving capabilities. It enables them to accurately articulate mathematical problems to AI, critically evaluate the logical soundness of AI-generated solutions, and ultimately transform AI output into personal mathematical understanding, thereby achieving deeper learning and cognitive advancement.

### 2.3 Cognitive tools

Cognitive tools refer to technological pathways specifically designed to extend and optimize learners’ cognitive processes. These tools are defined as computer applications capable of representing, organizing, or automating specific cognitive tasks, and forming an intellectual partnership with the learner (Lajoie and Derry, 1993). In mathematics learning environments, cognitive tools effectively reduce the cognitive load associated with understanding complex mathematical concepts through visualization, interactivity, and computational support, and fostering deeper conceptual understanding (Jonassen et al., 1998). Typical mathematical cognitive tools, such as MATLAB and the dynamic geometry software GeoGebra, demonstrate enhanced effects on mathematics learning across multiple dimensions (Mackenzie and Allen, 1998; Majid et al., 2013; Feng, 2024; Saha et al., 2010; Arbain and Shukor, 2015; Takači et al., 2015; Bekene Bedada and Machaba, 2022), and consistently enhance student learning motivation (Joshi, 2020). Research (Majid et al., 2013) indicates that MATLAB software, through multiple pathways such as visual demonstrations, mathematical computation support, and project-based practice, can effectively enhance students’ problem-solving abilities, foster positive mathematical attitudes and learning confidence, and exert a positive impact on mathematical achievement and learning motivation. The use of MATLAB in instruction has been shown to improve academic performance in advanced mathematics without imposing additional cognitive burden (Feng, 2024). And GeoGebra software aids students in constructing more intuitive conceptual models in topics such as calculus through its dynamic geometry and function graphing capabilities (Takači et al., 2015). Its use significantly boosts students’ confidence and conceptual understanding in learning calculus (Bekene Bedada and Machaba, 2022), while also markedly enhancing their positive perceptions of the learning process. By deeply integrating technological mediation with cognitive processes, cognitive tools provide robust support for online mathematics learning and enhance its overall effectiveness.

### 2.4 Perceived teacher support

Perceived teacher support refers to students’ subjective experience and evaluation of the emotional care, informational resources, instrumental assistance, and autonomy facilitation provided by teachers during their learning process. According to existing research, teacher support encompasses multiple dimensions, including emotional support (Patrick et al., 2007), informational support (Tardy, 1985), instrumental support (Bekene Bedada and Machaba, 2022), and autonomy support (Reeve and Cheon 2021). Supportive behaviors from teachers in academic or life contexts significantly promote students’ deep learning, with higher levels of teacher support correlating with richer positive experiences for students (Hao et al., 2021). Perceived teacher support shows a significant positive correlation with academic achievement (Zhang et al., 2019), and teachers’ supportive behaviors can effectively predict students’ academic performance (Tao et al., 2022).

As digital literacy increasingly becomes a core competency for learners, teacher support plays a significant role in boosting students’ confidence in applying technology and fostering their digital literacy development (Chen and Ma, 2023). In the fields of mathematics and science, teacher support has emerged as an important predictor of student literacy (Saroughi and Cheema, 2023). By providing informational resources, guidance on using technological tools, and encouraging student participation in highly interactive digital learning environments, teachers effectively promote the comprehensive development of students’ digital literacy (Wang, 2022). Supportive teacher behaviors, such as engaging in cross-curricular collaboration and pursuing professional development, can enhance students’ comprehensive competencies, including information technology literacy (Weidner, 2018). Simultaneously, perceived teacher support has also been proven to be a crucial factor influencing students’ online learning power, impacting aspects such as learning engagement (Yang and Du, 2025), self-directed learning capacity (Bai and Gu, 2022), self-regulation levels (Rui and Liu, 2023), and innovative problem-solving abilities (Hsia et al., 2021). A positive correlation exists between teacher support and learners’ information technology literacy (Chen and Ma, 2023). Effective dynamic resources in online learning can stimulate students’ positive achievement emotions (Pekrun, 2006), thus directly transforming their information technology literacy into comprehensive online learning power.

### 2.5 Research hypotheses

**Figure 1:**
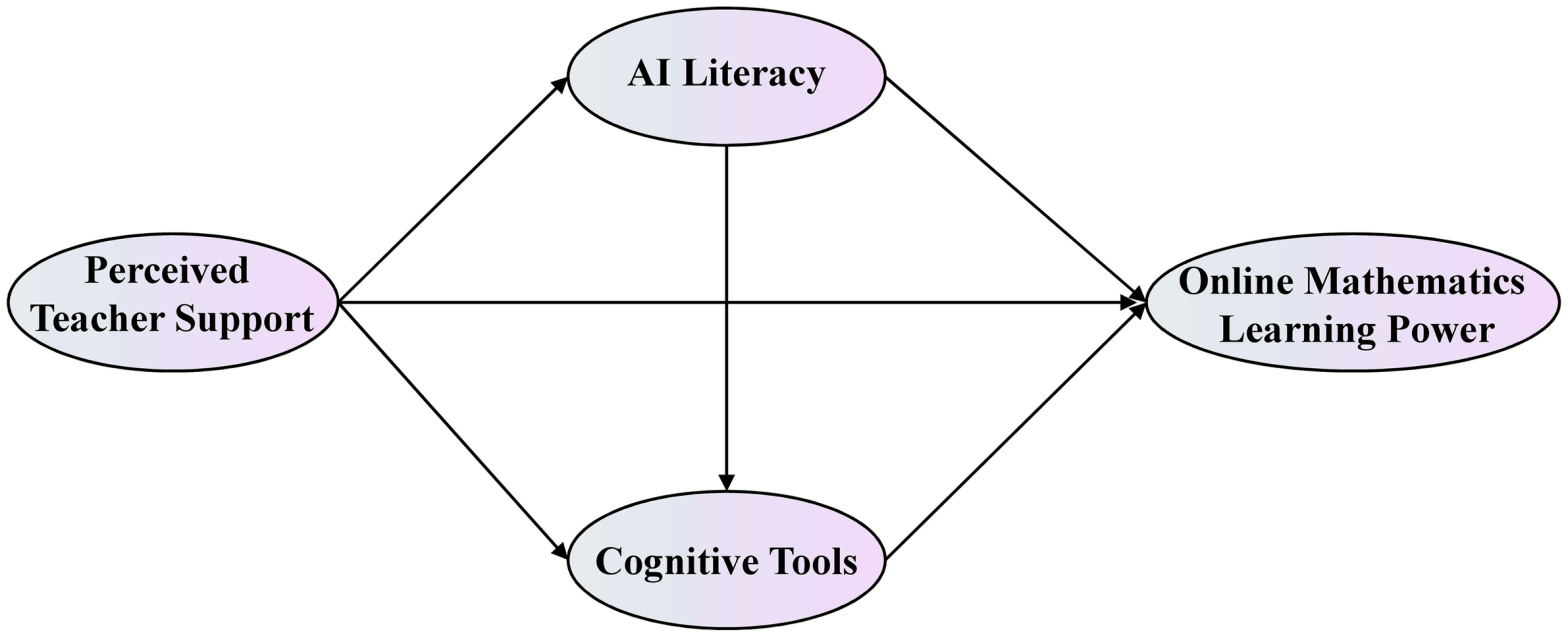
Conceptual model.

Based on the preceding theoretical foundation, this study constructs a mediating model (as illustrated in Figure 1). The model positions perceived teacher support as the independent variable and online mathematics learning power as the dependent variable, with a specific focus on examining the parallel mediating roles played by AI literacy and cognitive tools.

This model aims to examine the internal mechanism through which teacher support influences online mathematics learning power, specifically testing the extent to which this influence is realized by enhancing students’ AI literacy and promoting their use of cognitive tools. Accordingly, the following research hypotheses are proposed.

*H1*: Perceived teacher support positively predicts online mathematics learning power.

*H2*: Perceived teacher support positively predicts AI literacy.

*H3*: Perceived teacher support positively predicts cognitive tools.

*H4*: AI literacy positively predicts online mathematics learning power.

*H5*: AI literacy positively predicts cognitive tools.

*H6*: Cognitive tools positively predicts online mathematics learning power.

*H7*: AI literacy mediates the relationship between perceived teacher support and online mathematics learning power.

*H8*: Cognitive tools mediates the relationship between perceived teacher support and online mathematics learning power.

## 3 Methodology

### 3.1 Participants

This study was conducted at a comprehensive university in Eastern China. The participants were sophomore-year undergraduate students enrolled in online mathematics courses, including Advanced Mathematics, Probability and Statistics, and Linear Algebra. A total of 813 questionnaires were distributed. After screening and removing invalid responses, 758 valid questionnaires were retained, resulting in an effective response rate of 93.23%. Among the valid sample, 423 participants were male (55.8%) and 335 were female (44.2%). The entire survey process strictly adhered to the principle of voluntary participation and was conducted anonymously. All participants completed the questionnaire independently based on informed consent.

### 3.2 Measures

The study utilized a self-developed questionnaire comprising 27 items, all rated on a five-point Likert scale ranging from 1 (strongly disagree) to 5 (strongly agree). All scales were adapted from established instruments and modified to fit the online mathematics learning environment. To ensure content validity, the items were reviewed and revised by experts in the field of education. A pilot survey (*N* = 110) was then conducted for item analysis and reliability testing, after which the final scales were formed upon meeting all required criteria. For perceived teacher support (PTS), a 6-item scale was adapted from Lai (2015) and Pan (2022) and covered the dimensions of emotional support (1 item), behavioral support (2 items), technical guidance (1 item), and feedback support (2 items). For AI literacy (AI), a 5-item scale was modified from Wang et al. (2023) to assess technology application capabilities in mathematics learning contexts. For cognitive tools (CT), a 4-item scale was developed from the perspective of commonly used mathematics learning software and was designed to assess proficiency in data processing, computational programming, geometric visualization, and statistical applications. For online mathematics learning power (OMLP), a 12-item scale was developed by integrating theoretical frameworks from Claxton (2002), Crick (2006), and Crick et al. (2015) and encompassed the dimensions of adaptability, cognitive capacity, application capacity, and reflective capacity. All items are detailed in the Appendix.

Using SPSS 25.0, we performed the Kaiser-Meyer-Olkin (KMO) test of sampling adequacy and Bartlett’s test of sphericity (Kaiser, 1974) on the questionnaire items. The results showed a KMO value of 0.973, well above the recommended threshold of 0.90, and a significant Bartlett’s test of sphericity (χ^2^ = 22330.037, df = 351, *p* < 0.001). These indices collectively confirm that the data were highly suitable for factor analysis, thus providing a solid foundation for the subsequent confirmatory factor analysis (CFA).

Convergent validity is a key metric in confirmatory factor analysis (CFA) used to assess the effectiveness of scale measurement. A standardized factor loading of 0.70 is typically considered the minimum threshold for an item to effectively measure a latent variable (Hair et al., 2010). We conducted CFA on the four latent variables of perceived teacher support, AI literacy, cognitive tools, and online mathematics learning power using AMOS 21. The factor loading results for the observed items across all dimensions further confirmed the scales’ good convergent validity and reliability. The six items in the perceived teacher support dimension had loadings ranging from 0.765 to 0.921; the five items in the AI literacy dimension had loadings between 0.777 and 0.867; the four items in the cognitive tools dimension exhibited notably concentrated high loadings, distributed between 0.775 and 0.968; and the twelve items in the online mathematics learning power dimension also showed stable loadings in the range of 0.780 to 0.886. All item loadings substantially exceeded the 0.70 threshold, providing preliminary evidence of good convergent validity.

Table 1 presents the reliability and convergent validity test results for the four latent variables. The data indicate that the Cronbach’s *α* coefficients for perceived teacher support, AI literacy, cognitive tools, and online mathematics learning power are 0.951, 0.914, 0.937, and 0.968, respectively. All Cronbach’s α values exceed the recommended threshold of 0.70 (Fornell and Larcker, 1981; Nunnally and Bernstein, 1994), demonstrating excellent internal consistency reliability for each scale. In terms of convergent validity, the composite reliability (CR) values range between 0.918 and 0.967, all meeting the requirement of being greater than 0.7. The average variance extracted (AVE) values all surpass the critical threshold of 0.5, specifically 0.779, 0.691, 0.808, and 0.709, respectively, indicating good convergent validity for each variable (Fornell and Larcker, 1981) and confirming their effectiveness in measuring the corresponding latent constructs.

**Table 1:** Reliability and validity test results of the scales.

Latent variable	Number of items	Cronbach’s *α*	CR	AVE
PTS	6	0.951	0.955	0.779
AI	5	0.914	0.918	0.691
CT	4	0.937	0.944	0.808
OMLP	12	0.968	0.967	0.709

CR = composite reliability; AVE = average variance extracted.

Table 2 reports the descriptive statistics and correlation analysis results of the variables. The descriptive statistics show that the mean values of the variables range between 3.714 and 4.501, with perceived teacher support having the highest mean (4.501) and cognitive tools having the relatively lowest mean (3.714). The standard deviations range from 0.553 to 1.082, with cognitive tools exhibiting the largest standard deviation (1.082), while the standard deviations of the remaining variables are relatively close and small, indicating a relatively concentrated data distribution. The correlation analysis results reveal that all variables show significant positive correlations (*p* < 0.01), with correlation coefficients ranging from 0.488 to 0.760. Among them, perceived teacher support and AI literacy demonstrate high correlations with online mathematics learning power (*r* = 0.754, *r* = 0.760, respectively), providing preliminary correlational support for further investigation into their influence mechanisms.

**Table 2:** Descriptive statistics and correlation analysis of variables.

Latent variable	Mean	SD	1	2	3	4
1. PTS	4.501	0.556	—			
2. AI	4.446	0.582	0.721**	—		
3. CT	3.714	1.082	0.488**	0.521**	—	
4. OMLP	4.425	0.553	0.754**	0.760**	0.577**	—

*N* = 758. **Correlation is significant at the 0.01 level (two-tailed).

The measurement model of this study performs well on key metrics such as reliability, convergent validity, and discriminant validity. Meanwhile, the confirmatory factor analysis of the overall measurement model indicates that although the *χ*^2^/df value (4.99) is slightly elevated due to the sensitivity of the large sample size, other absolute and relative fit indices perform excellently (CFI = 0.943, TLI = 0.937, RMSEA = 0.073, and SRMR = 0.049). To assess common method bias, an unmeasured method factor was included in the confirmatory factor analysis. Comparison with the original model showed negligible fit index differences (ΔCFI = 0.001, ΔRMSEA = 0.001), both below established cut-offs (Cheung and Rensvold, 2002). The full model comparison results are provided in Supplementary Table S1. The results indicate that common method bias posed no serious threat to measurement validity.

## 4 Results

**Figure 2:**
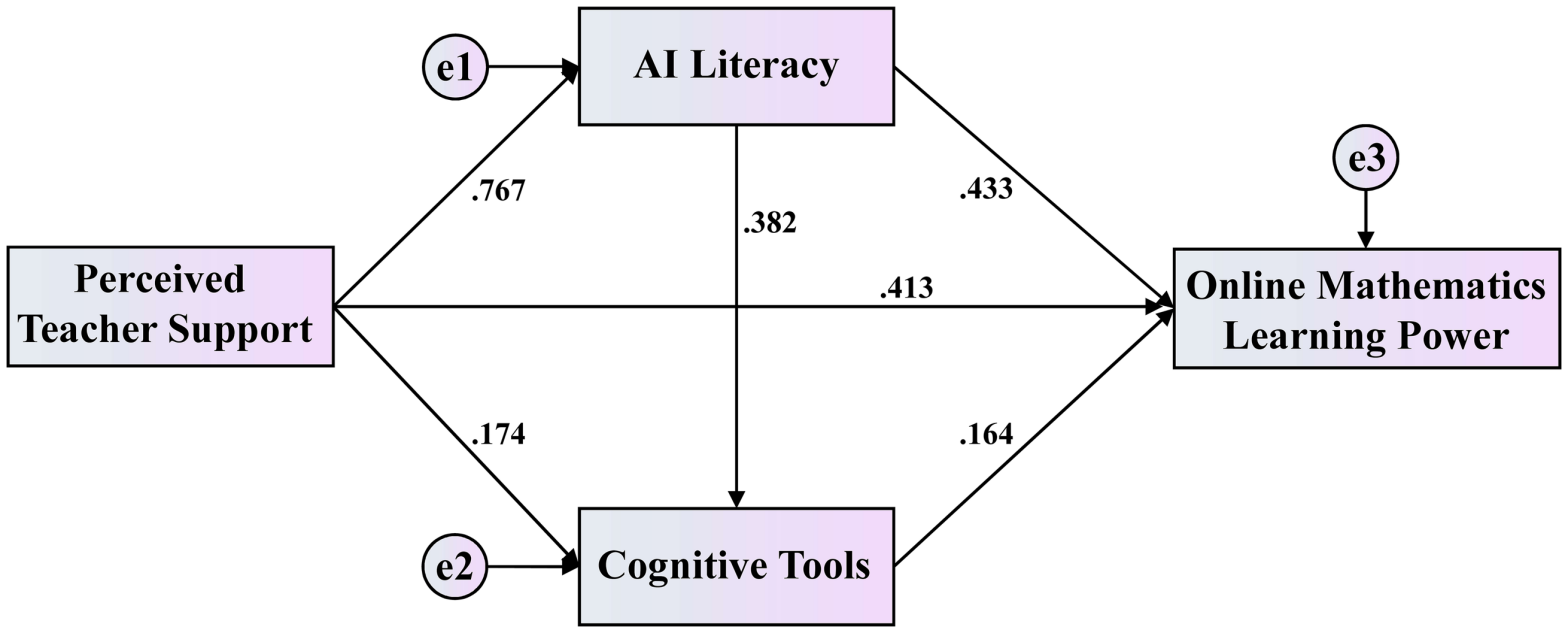
The final structural model with standardized estimates.

Structural equation modeling (SEM) was conducted using AMOS 21.0 to test the series of hypotheses regarding direct and indirect relationships among the variables. Using the bootstrap sampling method with 5,000 repeated subsamples and calculating 95% bias-corrected confidence intervals (*α* = 0.05), the final model (Figure 2) demonstrated good fit indices (*χ*^2^ = 1751.28; df = 608; *χ*^2^/df = 3.36; CFI = 0.96; TLI = 0.963; NFI = 0.912; RMSEA = 0.056). According to the joint cutoff criteria proposed by Hu and Bentler (1999), excellent model fit is indicated when both CFI and TLI exceed 0.95 and RMSEA is below 0.06. Furthermore, the final model explained 73.9% of the variance in online mathematics learning power (*R*^2^ = 0.739), demonstrating strong explanation.

Regarding the tests of direct effects, the standardized path analysis results in Table 3 show that all hypothesized direct effects were statistically significant (*p* < 0.01 or *p* < 0.001). Perceived teacher support had a significant positive direct effect on online mathematics learning power (*β* = 0.377, *p* < 0.001), supporting H1. Perceived teacher support also showed a significant positive direct effect on AI literacy (*β* = 0.767, *p* < 0.001), supporting H2, and a significant positive direct effect on the use of cognitive tools (*β* = 0.174, *p* < 0.01), supporting H3. AI literacy had a significant positive direct effect on online mathematics learning power (*β* = 0.433, *p* < 0.001), supporting H4, and a significant positive direct effect on the use of cognitive tools (*β* = 0.382, *p* < 0.001), supporting H5. Finally, the use of cognitive tools demonstrated a significant positive direct effect on online mathematics learning power (*β* = 0.164, *p* < 0.001), supporting H6.

**Table 3:** Standardized direct effects and hypothesis test results.

Path	Unstandardized estimate	Standardized estimate	S. E.	C. R.	*p*
PTS → AI	0.791	0.767	0.036	22.153	***
PTS → CT	0.402	0.174	0.128	3.138	**
AI → CT	0.856	0.382	0.128	6.685	***
PTS → OMLP	0.413	0.377	0.041	10.115	***
CT → OMLP	0.078	0.164	0.012	6.436	***
AI → OMLP	0.459	0.433	0.043	10.627	***

***p < 0.001; **p < 0.01; S.E. = standard error; C.R. = critical ratio.

**Figure 3:**
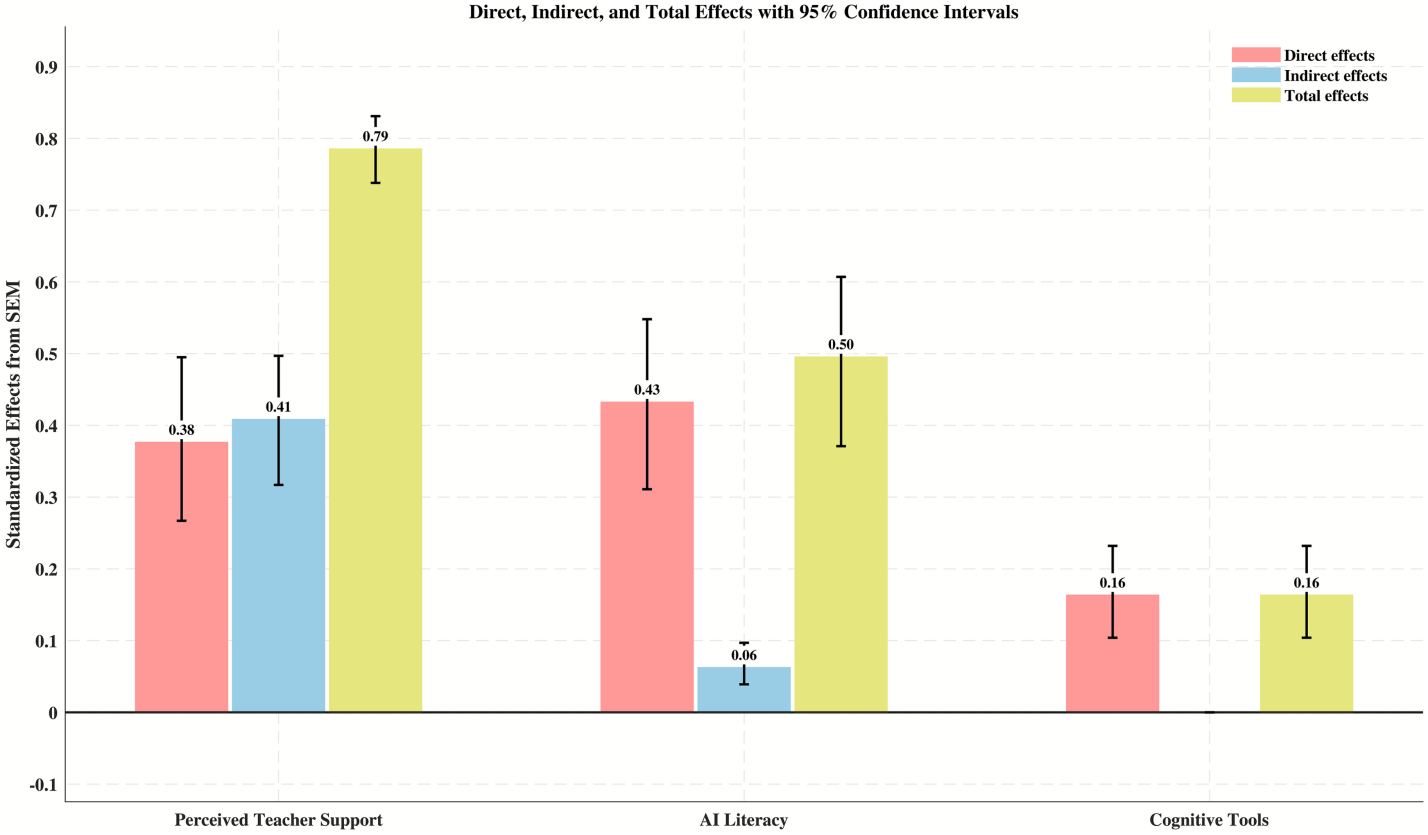
Standardized direct, indirect, and total effects of independent and mediating variables on online mathematics learning power, with error bars representing 95% confidence intervals.

The bootstrap test results for the mediation effects are presented in Table 4 and Figure 3. The 95% confidence intervals for all paths did not include zero (*p* < 0.001), indicating significant mediation effects. Perceived teacher support showed a significant direct effect of 0.377 [95% CI (0.267, 0.495)] and an indirect effect of 0.409 [95% CI (0.317, 0.497)] on online mathematics learning power, with a total effect reaching 0.786 [95% CI (0.738, 0.831)]. This indicates that teacher support is a key variable in online mathematics learning power. The influence of AI literacy on online mathematics learning power was primarily direct [0.433, 95% CI (0.311, 0.548)], while its indirect effect through cognitive tools was relatively small [0.063, 95% CI (0.039, 0.097)]. Cognitive tools exerted a significant direct effect of 0.164 [95% CI (0.104, 0.232)] on online mathematics learning power.

**Table 4:** Indirect (mediation) effect estimates and bootstrap confidence intervals.

Path	Effect type	*β* [95% CI]	*p*
PTS → OMLP	Direct	0.377 [0.267, 0.495]	< 0.001
	Indirect	0.409 [0.317, 0.497]	< 0.001
	Total	0.786 [0.738, 0.831]	< 0.001
AI → OMLP	Direct	0.433 [0.311, 0.548]	< 0.001
	Indirect	0.063 [0.039, 0.097]	< 0.001
	Total	0.496 [0.371, 0.607]	< 0.001
CT → OMLP	Direct	0.164 [0.104, 0.232]	< 0.001
	Indirect	—	—
	Total	0.164 [0.104, 0.232]	< 0.001

Bootstrap samples = 5,000. CI = confidence interval. Effects are standardized. Significant effects are indicated by a bootstrap CI that does not include zero.

The serial mediation analysis (see Table 5) examined the composition of the total indirect effect of perceived teacher support on online mathematics learning power (0.409, reported in Table 4). The independent mediating pathway via AI literacy (perceived teacher support → AI literacy → online mathematics learning power) contributed the largest portion, with an indirect effect of 0.332 [95% CI (0.235, 0.438)], accounting for 81.2% of the total indirect effect. This result supports H7. The independent mediating pathway *via* cognitive tools (perceived teacher support → cognitive tools → online mathematics learning power) showed a relatively smaller indirect effect of 0.029 [95% CI (0.009, 0.058)], accounting for 7.1%, which supports H8. Furthermore, the serial mediating pathway (perceived teacher support → AI literacy → cognitive tools → online mathematics learning power) had an indirect effect of 0.048 [95% CI (0.030, 0.076)], accounting for 11.7% of the total indirect effect.

**Table 5:** Indirect effects for each mediation path with bootstrap confidence intervals.

Path	*β* [95% CI]	*p*	% of Total indirect
PTS → AI → OMLP	0.332 [0.238, 0.441]	< 0.001	81.2%
PTS → CT → OMLP	0.029 [0.008, 0.056]	< 0.01	7.1%
PTS → AI → CT → OMLP	0.048 [0.027, 0.072]	< 0.001	11.7%

Bootstrap samples = 5,000. CI = confidence interval. Effects are standardized. Significant effects are indicated by a bootstrap CI that does not include zero.

To further analyze the specific contributions of each dimension of teacher support, we conducted separate regression analyses using the four support dimensions as independent variables to predict AI literacy and cognitive tools, respectively (see Table 6). For AI literacy, the four support dimensions collectively explained 52.0% of its variance. Among them, behavioral support (*β* = 0.301, *p* < 0.05) showed the strongest predictive effect and also had the largest unique explanatory power (*sr*^2^ = 0.015). Feedback support and emotional support followed in strength, while technical guidance exhibited the weakest predictive effect. This indicates that teachers’ behavioral support during students’ learning processes is the most critical supportive behavior for cultivating students’ AI literacy. For cognitive tools, the four support dimensions together accounted for 27.4% of the variance. Emotional support (*β* = 0.347, *p* < 0.05) had the strongest predictive effect, with its unique explanatory power (*sr*^2^ = 0.054) being substantially higher than that of the other variables. Technical guidance (*β* = 0.240, *p* < 0.001) also showed a significant predictive effect, whereas behavioral support and feedback support did not show significant predictions. These results suggest that emotional support is the core factor motivating students to use cognitive tools.

**Table 6:** Regression analysis predicting AI literacy and cognitive tools from teacher support dimensions.

Predictors	AI literacy		Cognitive tools	
	Std. *β*	*sr* ^2^	Std. *β*	*sr* ^2^
Emotional support	0.149***	0.010	0.347*	0.054
Behavioral support	0.301*	0.015	0.004	0.000
Technical guidance	0.120*	0.004	0.240***	0.015
Feedback support	0.209**	0.006	−0.022	0.000
Model summary	*R^2^* = 0.520, *F* = 204.20***		*R^2^* = 0.274, *F* = 70.90***	

***p < 0.001; **p < 0.01; *p < 0.05; Std. *β* = standardized regression coefficient; sr^2^ = squared semi-partial correlation.

In summary, all eight hypotheses were supported. The results confirm that perceived teacher support directly predicts online mathematics learning power, and also indirectly predicts it through both parallel and serial mediation involving AI literacy and cognitive tools. Together, these findings outline a mechanism through which teacher support contributes to online mathematics learning power: one direct, and another mediated sequentially by AI literacy and cognitive tools.

## 5 Discussion

This study confirms that perceived teacher support contributes significantly to online mathematics learning power through the mediating pathways of AI literacy and cognitive tools. The following sections discuss the key findings, theoretical contributions, and practical implications of this research within the established theoretical framework and in light of existing literature.

### 5.1 Key findings and interpretation

The findings indicate that perceived teacher support contributes to online mathematics learning power through a composite set of pathways. It exerts a direct effect on learning power (*β* = 0.377, *p* < 0.001), and its effect is also transmitted through both independent and sequential mediation via AI literacy and cognitive tools. The total indirect effect (0.409) slightly exceeds the direct effect. Further analysis revealed distinct contributions of different teacher support dimensions: behavioral support showed the strongest predictive effect on fostering students’ AI literacy, whereas emotional support emerged as the most critical factor in promoting the use of cognitive tools. These findings support the core propositions of social cognitive theory (Bandura, 1977) and are consistent with prior research in online learning (e.g., Chen and Ma, 2023; Saroughi and Cheema, 2023; Wang, 2022; Bai and Gu, 2022; Yang and Du, 2025). They confirm that external environmental support, specifically perceived teacher support, contributes directly to learning outcomes, while also contributing indirectly through the synergistic interplay of individual-internal competencies (AI literacy) and tool-application abilities (cognitive tool proficiency).

AI literacy and cognitive tools differ in their strength of contribution to online mathematics learning power. The direct effect of AI literacy (*β* = 0.433, *p* < 0.001) is larger than that of cognitive tools (*β* = 0.164, *p* < 0.001). Furthermore, the mediating pathway *via* AI literacy contributes the majority (81.2%) of the total indirect effect, exceeding the indirect contribution of the cognitive tools pathway (7.1%). This suggests that, in digital learning environments, students’ literacy in using AI for problem-solving holds greater importance than their skill in mastering specific cognitive tools. Additionally, AI literacy significantly and positively predicts cognitive tools (*β* = 0.382, *p* < 0.001), suggesting that students with higher AI literacy are more likely to use cognitive tools effectively.

The direct effect of perceived teacher support on cognitive tools is relatively limited (*β* = 0.174, *p* < 0.01). Its contribution to cognitive tools is largely mediated by AI literacy, with the serial mediation pathway accounting for 11.7% of the total indirect effect. This implies that in fostering students’ technology application, teacher support primarily acts by developing their internal AI literacy, which in turn contributes to more effective tool use, ultimately contributing to online mathematics learning power.

### 5.2 Theoretical implications

This study developed and validated a multiple mediation model encompassing “perceived teacher support → AI literacy/cognitive tools → online mathematics learning power”. Thereby, it extends the application of social cognitive theory to online mathematics learning environments and outlines the mechanism “external support contributing to learning outcomes through key digital competencies” in the intelligent era. Serial mediation analysis revealed that AI literacy not only exerts a strong direct predictive effect but also serves as a critical foundation for the use of cognitive tools. Furthermore, the research clarifies how specific dimensions of teacher support, particularly behavioral support and emotional support, make distinct contributions to these digital competencies: behavioral support is most predictive of AI literacy, whereas emotional support more strongly predicts cognitive tools. These findings enrich social support theory in online mathematics education and provide a theoretical framework for future research to examine how teacher support contributes to learning outcomes in different disciplinary contexts.

### 5.3 Practical implications

The findings of this study offer specific practical implications for online mathematics teaching in higher education.

Educators should adopt instructional strategies that integrate direct support, literacy cultivation, and tool guidance. During instruction, teachers should provide direct support through clear learning objectives, well-structured learning plans, and timely feedback. In the early stages of teaching, teachers should foster AI literacy through behavioral support by creating varied opportunities. For example, teachers can design well-guided AI tasks that enable students to use GenAI tools for mathematical problem-solving, model validation, and outcome evaluation. Teachers can also provide structured reflection templates to guide students in critically examining AI outputs and transforming them into personal mathematical understanding.

When students using cognitive tools or tackling advanced tasks such as programming, teachers should encourage them to actively utilize AI as a learning assistant. For instance, when students encounter difficulties while using MATLAB or GeoGebra, they can be encouraged to break down the problem, pose questions to AI tools such as code interpreters or conversational AI, and leverage AI to generate code snippets, debugging suggestions, or functional explanations. This approach helps lower technical barriers and enhances both confidence and efficiency in using cognitive tools.

Through this phased and targeted support, teachers can effectively promote the development of students’ key digital competencies, which in turn contributes to their online mathematics learning power.

## 6 Conclusion

This study constructed and tested a parallel multiple mediation model to examine the relationships among perceived teacher support, AI literacy, cognitive tools, and online mathematics learning power. The main conclusions are as follows.

First, the results confirm that perceived teacher support significantly and positively predicts online mathematics learning power, and it also predicts learning power indirectly through both independent and serial mediation pathways involving AI literacy and cognitive tools. Notably, the total indirect effect slightly exceeds the direct effect. This suggests that in technology-enhanced learning environments, the role of teacher support extends beyond direct instruction; it also contributes to online mathematics learning power by fostering students’ AI literacy and facilitating their effective use of cognitive tools.

Second, AI literacy and cognitive tools play distinct roles within the mechanism. AI literacy demonstrates a stronger predictive capacity, exhibiting both a significant direct predictive effect on online mathematics learning power and a substantial mediating role. Furthermore, AI literacy significantly contributes to proficiency with cognitive tools, indicating that higher levels of AI literacy are associated with more effective use of these tools.

Third, the specific contributions of different teacher-support dimensions clarify actionable pathways for educators: behavioral support is most effective for cultivating students’ AI literacy, while emotional support is most critical for building confidence in using cognitive tools.

Based on these findings, this study recommends that online mathematics instruction integrate direct support, AI literacy cultivation, and cognitive-tool guidance to promote the development of students’ online mathematics learning power.

This study has several limitations that point to future research directions. The data were self-reported, and the sample was drawn from a single cultural and institutional context (universities in eastern China), which may restrict the generalizability of the findings. Moreover, the conceptualization of AI literacy was relatively focused on the mathematics-learning context and did not encompass broader dimensions of the construct; future research could extend the investigation within a more comprehensive literacy framework, including ethical and socio-cultural dimensions. Subsequent work could also employ multi-group comparisons, longitudinal designs, or the inclusion of moderators (e.g., self-efficacy) to further refine the understanding of the mechanisms underlying online mathematics learning power.

Despite these limitations, this study offers initial insights into how external environmental factors (teacher support) and student digital competencies (AI literacy, cognitive tools) jointly contribute to online mathematics learning power. It thereby provides both a theoretical foundation and practical guidance for online mathematics instruction in the intelligent era.

## Data Availability

The raw data supporting the conclusions of this article will be made available by the authors, without undue reservation.

## Appendix: Measures

**Table:**

Item	Factor loadings
Perceived teacher support. Adopted from Lai (2015) and Pan (2022). *α* = 0.951	
Encourage use of online learning tools (e.g., course platforms, AI tools)	0.765
Recommend useful online learning resources	0.888
Assign clear learning tasks (e.g., lecture slides, videos, exercises, assignments)	0.904
Use technological tools to visualize abstract mathematics or solve problems	0.888
Provide timely feedback on learning performance	0.921
Regularly monitor online learning progress	0.921
AI Literacy. Adopted from Wang et al. (2023). *α* = 0.914	
Skillfully utilize common AI tools (e.g., Kimi, Doubao) to facilitate mathematics learning	0.817
Select and combine appropriate AI tools to improve efficiency	0.777
Effectively pose questions to AI tools to obtain precise, high-quality information and solutions	0.858
Critically evaluate the reliability, accuracy, and potential biases of AI-generated content	0.834
Use AI tools to deepen understanding of math concepts and expand problem-solving approaches	0.867
Cognitive Tools. *α* = 0.937	
Use Excel/WPS for basic data analysis (e.g., creating data charts)	0.775
Use Python/Matlab for mathematical computations (e.g., solving equations, matrix operations)	0.916
Use GeoGebra to explore geometric problems (e.g., function graphs, geometric transformations)	0.925
Use SPSS/Python/Matlab for basic statistical analysis (e.g., means, correlation analysis)	0.968
Online mathematics learning power. *α* = 0.968	
Overcome environmental distractions and maintain focus during mathematics learning	0.849
Quickly adapt to various online learning platforms (e.g., Zhihuishu, Rain Classroom, Bilibili)	0.849
Proactively seek solutions when encountering network or platform issues	0.863
Integrate online resources (e.g., videos, question banks) to solve mathematical exercises	0.857
Evaluate the accuracy of online mathematics learning resources	0.880
Deepen understanding of mathematical concepts through discussions	0.844
Consolidate foundational mathematical knowledge using online resources	0.884
Independently complete math assignments or exercises using online course resources	0.886
Apply mathematical knowledge to discipline-specific problems	0.780
Regularly self-assess mathematics learning outcomes	0.812
Adjust learning priorities based on online quiz results	0.798
Reflect on problem-solving approaches through discussions with peers	0.793
