# Supplementary material for: The effects of perceived teacher support on online mathematics learning power: the mediating roles of artificial intelligence literacy and cognitive tools
Source: Front Psychol. 2026 Feb 24;17:1763924. doi: 10.3389/fpsyg.2026.1763924 (PMC12971719; doi:10.3389/fpsyg.2026.1763924)
Supplement: Supplementary file 1 [file Table_S1.DOCX]

Supplementary Material

# Supplementary Table S1

Table S1. Model Fit Indices for the CFA and the CFA‑CMV Models

| Model | χ² | df | χ²/df | CFI | RMSEA | SRMR |
| --- | --- | --- | --- | --- | --- | --- |
| Four-factor CFA Model | 1586.7 | 318 | 4.99 | 0.943 | 0.073 | 0.049 |
| Five-factor CFA-CMV Model | 1556.8 | 317 | 4.91 | 0.944 | 0.072 | 0.232 |
